# Cognitive Correlates of Borderline Personality Disorder Features in Youth with Bipolar Spectrum Disorders and Bipolar Offspring

**DOI:** 10.3390/brainsci15040390

**Published:** 2025-04-10

**Authors:** Alessio Simonetti, Sherin Kurian, Evelina Bernardi, Antonio Restaino, Francesca Bardi, Claudia Calderoni, Gabriele Sani, Jair C. Soares, Kirti Saxena

**Affiliations:** 1 Department of Psychiatry and Behavioral Sciences, Baylor College of Medicine, Houston, TX 77030, USA; alessio.simonetti@policlinicogemelli.it (A.S.); sherin.kurian@bcm.edu (S.K.); 2 Section of Psychiatry, Department of Neuroscience, Head-Neck and Chest, Fondazione Policlinico Universitario Agostino Gemelli IRCCS, 00168 Rome, Italy; gabriele.sani@unicatt.it; 3 Department of Child and Adolescent Psychiatry, Texas Children’s Hospital, Houston, TX 77030, USA; 4 Section of Psychiatry, Department of Neuroscience, Head-Neck and Chest, Università Cattolica del Sacro Cuore, 00193 Rome, Italy; evelina.bernardi@unicatt.it (E.B.); antonio.restaino@unicatt.it (A.R.); francesca.bardi97@gmail.com (F.B.); claudia.calderoni1991@gmail.com (C.C.); 5 Department of Psychiatry and Behavioral Sciences, University of Texas Health Science Center, Houston, TX 77030, USA; jair.c.soares@uth.tmc.edu

**Keywords:** pediatric bipolar disorder, bipolar offspring, borderline personality disorder, cognition

## Abstract

**Background:** Bipolar disorder (BD) and borderline personality disorder (BPD) share common cognitive impairments. These deficits are also shared by bipolar offspring (BD-OFF). Nevertheless, little is known regarding the association between cognitive impairments and BPD features in youth with BD and BD-OFF. **Objectives:** This study aimed to investigate the association between BPD features and cognitive impairments in youth with BD and BD-OFF. **Methods:** Thirty-nine participants (7–17 years) with BD, 18 BD-OFF, and 50 healthy controls (HCs) were recruited. BPD features were assessed using the Borderline Personality Features Scale for Children (BPFS-C). Deficits in executive functions and affective processing were assessed using tasks from the Cambridge Neuropsychological Test Automated Battery (CANTAB), namely, the Cambridge gambling task (CGT), the stockings of Cambridge (SOC), and the Affective Go/No-Go (AGN) and rapid visual processing (RVP) tasks. Between-group differences were analyzed through ANOVAs. Relationships between the BPFS-C and cognitive tasks were examined using multiple linear regressions in youth with BD and BD-OFF. **Results:** Youth with BD and BD-OFF showed higher scores on the BPFS-C. Youth with BD had increased deficits in the CGT and SOC compared to HCs. In both youth with BD and BD-OFF, BPD features were associated with increased deficits in the CGT, and a bias toward positive emotions in the AGN task. **Conclusions:** In youth with BD and BD-OFF, clinical and cognitive assessments for BPD features are of relevance as they have the potential to inform targeted interventions.

## 1. Introduction

Bipolar disorder (BD) and borderline personality disorder (BPD) are frequently comorbid, as both share traits such as impulsivity, emotion dysregulation, suicidality, self-injurious behaviors, and substance abuse [1].

A meta-analysis of 42 studies by Fornaro et al. [2] revealed that 21.6% of adults with BD also met the Diagnostic and Statistical Manual of Mental Disorders (DSM) criteria for BPD, and 18.5% of adults with BPD met DSM criteria for BD. In a review by Zimmerman and Morgan [3], 10% of adults with BPD had bipolar I disorder (BDI), 10% had bipolar II disorder (BDII), and 10% of adults with BDI and 20% of individuals with BDII also had BPD. Individuals with BD and a comorbid personality disorder show high rates of suicide attempts, psychiatric hospitalizations, and overall functional impairment [4,5].

The pathophysiology of BD and BPD appears to overlap, as both disorders share deficits involving emotion regulation, cognition, or both [6]. Specifically, emotion dysregulation appears to be one of the most important features of BD and BPD [7]. When compared with healthy controls (HCs), adults with BPD and BD had in common decreased performance in executive function subdomains, i.e., planning and problem-solving [8,9,10,11,12,13]. On the other hand, in adults with BPD, deficits related to planning and difficulty attending to specific cues were more prevalent, whereas deficits related to processing speed and strategy formation were specific to adults with BD. Furthermore, adults with BPD appear to perform worse in attentional tasks [14,15,16,17], whereas deficits in executive functions appear to be most consistently observed across all mood states of BD [18].

The profile of cognitive deficits in youth with BD closely resembles that found in the adult form of the disorder [19,20,21], suggesting that many of the deficits observed in adults with BD are present earlier in the illness course. Cognitive deficits in youths with BD include impairments in sustained attention, verbal memory, learning and planning, and visuospatial skills [22]. Additionally, bipolar offspring (BD-OFF) have shown cognitive and emotion regulation deficits in areas such as attention, processing speed, executive functions, and emotion regulation [23,24,25,26,27,28,29,30].

Even though there are studies providing insight into possible shared mechanisms in subjects with BD and BD-off, there is a lack of specific studies investigating cognitive impairments in youth with BD and BD-OFF with comorbid BPD. Understanding cognitive alterations underlying maladaptive personality traits in BD early on is clinically relevant as behaviors related to these traits lead to an increase in risky, aggressive behaviors and legal problems [31]. This is significant, as it directly impacts targeted treatment interventions. Notably, BPD is highly responsive to dialectical behavior therapy, whereas medication management and family-focused therapy are the standard treatment for managing symptoms of BD [32]. Furthermore, a careful evaluation of cognitive impairments associated with borderline features in youth with BD and BD-OFF is of significance to identify specific at-risk youth, in order to target specific treatment interventions.

The current pilot study sought to investigate the relationship between cognitive impairments and borderline personality features in youth with BD and bipolar offspring (BD-OFF). We assessed borderline personality features using the Borderline Personality Features Scale-Child (BPFS-C) and selected tasks from the Cambridge Neuropsychological Test Automated Battery (CANTAB) for executive functions, affective processing, and sustained attention. In youth with BD and BD-OFF, we correlated borderline personality features with the selected cognitive tasks. We anticipated that youth with BD will show greater impairment in executive functions, affective processing, and sustained attention than HCs. We anticipated the same cognitive impairments in BD-OFF, however, with decreased severity. In youth with BD and BD-OFF, higher scores on the BPFS-C were expected to correlate with greater cognitive impairments in executive functions, affective processing, and sustained attention.

## 2. Materials and Methods

### 2.1. Study Participants

This is a cross-sectional study. This study received ethical approval from the Institutional Review Board of the Baylor College of Medicine (BCM). Eligibility criteria included the following: age between 7 and 17 years; a diagnosis of bipolar spectrum disorders, i.e., BDI, BDII, and BD not otherwise specified (only for youth with BD); BD-OFF needed to be offspring of a biological parent with BD; BD-OFF were also required to not have a diagnosis of BD; and HCs needed to show no psychiatric or neurological disorders. Exclusion criteria included schizophrenia, attention-deficit hyperactivity disorder (ADHD) without comorbid BD, anxiety disorders without comorbid BD, a current substance use disorder, intellectual disability, autism spectrum disorder, and severe neurological or organic disorders. Participants were enrolled through the child and adolescent psychiatry outpatient clinic at Texas Children’s Hospital. Recruitment materials were distributed across hospital clinics and online platforms to enroll youth with BD, BD-OFF, and HCs. Parents or legal guardians of eligible youth, deemed eligible following an initial phone-based screening, were contacted for participation. Members of the research team delivered a comprehensive explanation of the aims and procedures of the study, and written informed consent was obtained from a parent or legal guardian prior to conducting any research-related procedures.

### 2.2. Clinical Assessments

Study participants underwent evaluation using the 7.0.1 version of the Mini International Neuropsychiatric Interview–Kid screen (MINI-KID), the parent MINI-KID, and the Wechsler Abbreviated Scale of Intelligence-II (WASI-II) [33,34]. The MINI-KID and parent MINI-KID are structured diagnostic tools commonly employed to identify psychiatric conditions and have been revised to align with DSM-5 diagnostic criteria. The WASI–II was administered to estimate overall cognitive ability, providing an age- and sex-adjusted composite intelligence quotient (I.Q.) score.

A board-certified psychiatrist conducted the psychiatric assessments using DSM-5 criteria to establish the presence of BDI and BDII. For the diagnosis of BD not otherwise specified (BD-NOS), the Course and Outcome of Bipolar Youth (COBY) research criteria were utilized [35]. COBY criteria tackle the high heterogeneity of criteria for subthreshold BD [35,36]. Research indicates that young people diagnosed with BD-NOS using the COBY criteria share multiple clinical characteristics with BDI, and frequently exhibit longitudinal progression to BDI or BDII diagnoses [37].

### 2.3. Psychiatric Symptoms

The severity of depressive symptoms was evaluated using the clinician-administered 17-item Children’s Depression Rating Scale–Revised (CDRS-R) [38]. Scoring thresholds for the CDRS-R are interpreted as follows: absence/minimal depressive symptoms (0 to 28), subclinical/borderline symptoms (29–39), and clinically significant depression (≥40). The severity of manic symptoms was assessed via the clinician-administered Young Mania Rating Scale (YMRS) [39]. Interpretive guidelines for the YMRS are as follows: no evidence of manic symptoms (0–12), minimal manic symptoms (13–20), mild manic symptoms (20–26), moderate manic symptoms (26–38), and severe manic symptoms (>38). Borderline personality features were assessed using the BPFS-C [40], a self-report instrument. The BPFS-C comprises 24 items covering four core domains: (1) affective instability (AI); (2) identity problems (IPs); (3) negative relationships (NRs); (4) self-harm (SH). A total score ≥ 60 is suggestive of the presence of borderline personality disorder, while subscores ≥ 15 on any domain are considered indicative of clinically relevant psychopathology.

### 2.4. Cognitive Testing

Cognitive evaluations were assessed using a set of tasks from the CANTAB, a widely validated computerized assessment tool [41]. The targeted cognitive domains assessed in this study included the following: affective processing, executive functions, and sustained attention. These tests were chosen as these domains were frequently found to be altered in youth with BD, BD-OFF, and those with borderline personality disorder [6,7,8,9,10,11,12,13,14,15,16,17,18]. A brief description of the CANTAB tasks used is provided below.

#### 2.4.1. Affective Processing

Affective processing was examined using the Affective Go/No-Go (AGN) task, which evaluates information processing biases toward positive and negative stimuli. During the test, participants are shown a series of stimulus blocks on a computer screen, each containing words from three distinct affective categories: positive, negative, and neutral. At the beginning of each block, a target emotional category (e.g., positive) is indicated, and participants are instructed to respond only to stimuli that correspond to that category. They must then select words that are congruent with the designated valence (e.g., respond to positive words when positive is the target). Key outcome measures include reaction times (RTs) for positive (AGN-RTs-positive) and negative (AGN-RTs-negative) words, commission errors (CEs) for positive (AGN-CEs-positive) and negative (AGN-CEs-negative) words, and omissions for positive (AGN-omissions-positive) and negative (AGN-omissions-negative) words.

#### 2.4.2. Executive Functions

Executive functions were assessed through two CANTAB tasks: the Cambridge gambling task (CGT) and the stockings of Cambridge (SOC).

The CGT is designed to evaluate risk-based decision making and impulsivity in contexts independent of reinforcement learning. During the task, participants view a horizontal array of ten red and blue boxes on a screen. A yellow token is randomly concealed within one of these boxes. Using on-screen “Red” and “Blue” selection buttons, participants must indicate which color they believe is more likely to contain the hidden token. They start with a fixed number of points and are required to place a bet, wagering a proportion of their points based on their choice. A central circle displays the current bet value, which fluctuates incrementally. Participants must confirm their wager when the displayed amount matches their desired proportion. Depending on whether the participant correctly identifies the token’s location, the wagered amount is either added to or subtracted from their score.

Outcome measures derived from the CGT include the following: delay aversion (CGT-delay aversion), deliberation time (CGT-deliberation time), proportion bet (CGT-proportion bet), quality of decision making (CGT-quality of decision making), risk adjustment (CGT-risk adjustment), and risk taking (CGT-risk taking).

The SOC is a test of spatial planning and goal-directed problem-solving. In this task, participants are shown two displays, each displaying three vertical stockings suspended from a horizontal bar, with colored balls arranged differently in each display. The objective is to reorganize the balls in the lower display to match the target arrangement in the upper display, using the fewest possible moves. The main performance outcome is the number of problems successfully solved using the minimum number of moves (SOC-moves).

#### 2.4.3. Sustained Attention

Sustained attention was measured using the rapid visual information processing (RVP) task. During this assessment, participants are shown a white central box on the screen in which digits ranging from 2 to 9 are displayed in a pseudo-randomized sequence at a rate of 100 digits per minute. Individuals are instructed to monitor the stream of numbers for specific target sequences (e.g., 2-4-6, 3-5-7, 4-6-8). Upon detecting a target sequence, participants must promptly respond by pressing a designed central response button. The task’s complexity is adjusted by varying the number of simultaneous target sequences, ranging from single- to triple-sequence detection. Primary outcome measures cover A, a signal detection measure evaluating sensitivity to the target sequence independent of response bias (RVPA), the speed of response (RVP-mean latency), the probability of false alarms (RVP-probability of false alarms), and the probability of a hit (RVP-probability of hit).

### 2.5. Statistical Analysis

#### 2.5.1. Demographic and Clinical Characteristics

Group differences in demographic and clinical characteristics were examined using one-way factorial analyses of variance (ANOVAs) for continuous variables (age, education years, intelligence quotient (I.Q.), CDRS-R, and YMRS total scores), and chi-square tests for nominal variables (gender, race/ethnicity, type of comorbidity, and current pharmacotherapy).

#### 2.5.2. Cognitive Characteristics and Borderline Personality Features

One-way factorial ANOVAs were performed in participants who completed the BPFS to investigate intergroup differences in cognition and borderline personality features. In these analyses, the groups (BD, BD-OFF, and HCs) served as independent variables, while the scores from the CANTAB tasks, BPFS subscales (BPFS-AI; BPFS-IPs; BPFS-NRs; BPFS-SH), and BPFS total scores (BPFS-total) were treated as dependent variables. To further analyze significant effects, Bonferroni-corrected post hoc comparisons were applied, with a threshold for statistical significance set at *p* < 0.05.

#### 2.5.3. Relationship Between Borderline Personality Features and Cognitive Characteristics

In order to explore the relationship between borderline personality features and cognitive characteristics, multiple linear regression analyses were conducted. In each model, BPFS subscale scores (BPFS-AI; BPFS-IPs; BPFS-NRs; BPFS-SH) and BPFS total scores (BPFS-total) were used as predictor variables, while scores from individual CANTAB tasks served as dependent variables. Statistical significance was determined using a *p*-value threshold of <0.05. To examine the potential interaction effect between the diagnosis of BD and BD-OFF, ANOVAs were performed. In these models, an interaction term between each BPFS subscale and diagnostic group (BD or BD-OFF) was included as the independent variable, and CANTAB task performance was used as the outcome variable.

## 3. Results

### 3.1. Demographic and Clinical Characteristics

The study sample included 107 subjects: 39 youth with BD, 18 BD-OFF, and 50 HCs. The groups differed in years of education, I.Q., CDRS and YMRS mean scores, comorbidities, and the number of psychotropic drugs. Differences are shown in Table 1.

### 3.2. Cognitive Characteristics

One-way ANOVAs showed significant differences in CGT-deliberation time and SOC-moves. Post hoc analyses showed longer deliberation time in the CGT in youth with BD than in HCs. As regards SOC-moves, those with BD solved fewer problems in the minimum number of moves than HCs. No other differences emerged (see Table 2).

### 3.3. Borderline Personality Features

ANOVAs revealed significant differences in BPFS-AI, BPRS-IP, BPFS-NR, BPFS-SH, and BPFS-total scores. Post hoc analyses showed higher scores in BPFS-AI, BPRS-IPs, BPFSs-NR, BPFS-SH, and BPFS-total in youth with BD than HCs; BD-OFF showed higher scores in BPFS-AI, BPFS-NR, BPFS-SH, and BPFS-total than HCs (see Table 3).

### 3.4. Relationship Between Borderline Personality Features and Cognition

Linear regression analyses revealed that in BD and BD-OFF, BPFS-IPs negatively predicted CGT-delay aversion, and positively predicted CGT-overall proportion bet and CGT-risk taking. BPFS-NRs negatively predicted AGN-RTs-positive. BPFS-SH was a negative predictor of AGN-RTs-positive and CGT-quality of decision making; BPFS-total negatively predicted AGT-RTs-positive and positively predicted CGT-overall proportion bet and CGT-risk taking.

All other correlations were non-significant (see Table 4, Table 5, Table 6 and Table 7). No interactions emerged.

### 3.5. Effect of Possible Confounding Variables

Since the groups differed in terms of years of education, I.Q., depressive and manic symptoms, number of comorbidities, and pharmacotherapy, the results of the present study were controlled for the effect of these variables. Specifically, analyses of covariance (ANCOVAs) were performed in order to control differences in cognitive testing. All the aforementioned variables entered the ANCOVAs as covariates, whereas groups and CANTAB subtests were independent and dependent variables. Main and interaction effects were evaluated. No main effects or interactions emerged.

**Table 2:** Performances on CANTAB tests of 39 youth with BD, 18 BD-OFF, and 50 HCs.

	BD (N = 39)	BD-OFF (N = 18)	HCs (N = 50)	χ^2^/F	*p*-Value	HC vs. BD *p*-Value	HC vs. BD d	HC vs. BD-OFF *p*-Value	HC vs. BD-OFF d	BD vs. BD-OFF *p*-Value	BD vs. BD-OFF d
AGN-RTs-positive (mean ± SD)	5.27 ± 9.76	4.65 ± 1.08	5.24 ± 1.32	1.67	0.194	1.000	0.007	0.288	0.339	0.272	0.787
AGN-RTs-negative (mean ± SD)	4.99 ± 1.24	4.90 ± 1.26	4.82 ± 1.19	0.19	0.824	1.000	0.063	1.000	0.248	1.000	0.302
AGN-CEs-positive (mean ± SD)	5.30 ± 4.28	5.59 ± 3.89	4.71 ± 3.88	0.41	0.666	1.000	0.399	1.000	0.153	1.000	0.233
AGN-CEs-negative (mean ± SD)	6.09 ± 4.74	6.81 ± 4.23	4.93 ± 4.09	1.38	0.258	0.690	0.378	0.426	0.119	1.000	0.232
AGN-omissions-positive (mean ± SD)	6.74 ± 5.60	4.34 ± 3.27	5.07 ± 5.08	1.71	0.187	0.406	0.183	1.000	0.210	0.348	0.042
AGN-omissions-negative (mean ± SD)	7.07 ± 6.13	4.84 ± 3.43	5.16 ± 5.27	1.62	0.203	0.331	0.110	1.000	0.253	0.509	0.148
CGT-delay aversion (mean ± SD)	0.53 ± 0.29	0.61 ± 0.17	0.59 ± 0.21	1.08	0.344	0.060	0.122	1.000	0.167	0.738	0.041
CGT-deliberation time (mean ± SD)	3350.90 ± 1900.63	3059.4 ± 1195.63	2423.70 ± 901.90	4.76	**0.011**	**0.010**	0.292	0.341	0.449	1.000	0.124
CGT-overall proportion bet (mean ± SD)	0.56 ± 0.16	0.52 ± 0.09	0.51 ± 0.13	1.45	0.240	0.296	0.074	1.000	0.239	0.956	0.153
CGT-quality of decision making (mean ± SD)	0.90 ± 0.10	0.88 ± 0.09	0.86 ± 0.13	1.04	0.356	0.456	0.066	1.000	0.146	1.000	0.074
CGT-risk adjustment (mean ± SD)	0.26 ± 0.99	0.31 ± 0.61	0.54 ± 0.91	1.04	0.357	0.521	0.228	1.000	0.190	1.000	0.076
CGT-risk taking (mean ± SD)	0.58 ± 0.16	0.56 ± 0.11	0.54 ± 0.14	0.77	0.466	0.653	0.138	1.000	0.275	1.000	0.133
RVPA (mean ± SD)	0.80 ± 0.07	0.83 ± 0.06	0.84 ± 0.06	2.65	0.077	0.073	0.117	1.000	0.261	0.729	0.141
RVP-mean latency (mean ± SD)	618.76 ± 197.69	620.41 ± 231.74	558.63 ± 147.98	1.01	0.368	0.642	0.153	0.869	0.569	1.000	0.423
RVP-probability of false alarms (mean ± SD)	0.04 ± 0.08	0.04 ± 0.07	0.02 ± 0.05	0.85	0.433	0.720	0.329	1.000	0.200	1.000	0.164
RVP-probability of hit (mean ± SD)	0.37 ± 0.18	0.43 ± 0.22	0.45 ± 0.19	1.33	0.272	0.334	0.105	1.000	0.45	1.000	0.368
SOC-moves (mean ± SD)	5.85 ± 1.88	7.06 ± 1.66	6.64 ± 1.99	3.12	**0.048**	**0.047**	0.549	1.000	0.174	0.094	0.341

Note: significant *p*-values (*p* < 0.05) are indicated in **bold**. AGN—Affective Go/No-Go; BD—youth with bipolar disorder; BD-OFF—offspring of subjects with BD; CANTAB—Cambridge Neuropsychological Test Automated Battery; CEs—commission errors; CGT—Cambridge gambling task; HC(s)—healthy control(s); RTs—reaction times; RVP—rapid visual information processing; RVPA—rapid visual information processing response tendency; SD—standard deviation; SOC—stockings of Cambridge.

**Table 3:** Scores on BPFS of 39 youth with BD, 18 BD-OFF, and 50 HCs.

	BD (N = 39)	BD-OFF (N = 18)	HCs (N = 50)	χ^2^/F	*p*-Value	HC vs. BD *p*-Value	HC vs. BD d	HC vs. BD-OFF *p*-Value	HC vs. BD-OFF d	BD vs. BD-OFF *p*-Value	BD vs. BD-OFF d
BPFS-AI (mean ± SD)	19.31 ± 4.35	16.50 ± 5.58	12.50 ± 3.30	30.06	**<0.001**	**<0.001**	1.764	**0.002**	2.399	0.057	3.559
BPFS-IPs (mean ± SD)	13.79 ± 4.48	12.56 ± 4.62	10.98 ± 4.39	4.41	**0.015**	**0.012**	0.633	0.605	0.351	0.996	0.270
BPFS-NRs (mean ± SD)	15.44 ± 4.97	14.06 ± 5.39	10.02 ± 3.88	16.39	**<0.001**	**<0.001**	1.215	**0.005**	0.860	0.874	0.266
BPFS-SH (mean ± SD)	16.31 ± 5.74	14.78 ± 5.55	10.82 ± 3.32	15.63	**<0.001**	**<0.001**	0.171	**0.009**	0.866	0.773	0.271
BPFS-TOT (mean ± SD)	64.85 ± 16.24	57.89 ± 17.65	44.32 ± 12.09	22.08	**<0.001**	**<0.001**	1.430	**0.003**	0.897	0.299	0.410

Note: significant *p*-values (*p* < 0.05) are indicated in **bold**. AI—affective instability; BD—youth with bipolar disorder; BD-OFF—offspring of subjects with BD; BPFS—Borderline Personality Features Scale; HCs—healthy controls; IPs—identity problems; NRs—negative relationships; SD—standard deviation; SH—self-harm; TOT—total.

**Table 4:** Linear regressions of BPFS subscales and total scores and Affective Go/No Go task in 39 youth with BD and 18 BD-OFF.

	AGN-RTs-Positive			AGN-RTs-Negative			AGN-CEs-Positive		
	β	*t*	*p*-Value	β	*t*	*p*-Value	β	*t*	*p*-Value
BPFS-AI	−0.165	−1.141	0.260	0.242	1.678	0.100	−0.105	−0.740	0.463
BPFS-IPs	−0.171	−1.230	0.0225	0.154	1.074	0.288	−0.124	−0.906	0.370
BPFS-NRs	−0.284	−2.047	**0.047**	0.185	1.284	0.205	−0.074	−0.528	0.600
BPFS-SH	−0.405	−2.814	**0.007**	0.198	1.330	0.190	−0.133	−0.920	0.362
BPFS-TOT	−0.320	−2.293	**0.027**	0.237	1.647	0.106	−0.134	−0.952	0.346
	**AGN-CEs-Negative**			**AGN-Omissions-Positive**			**AGN-Omissions-Negative**		
	**β**	***t***	***p*-Value**	**β**	***t***	***p*-Value**	**β**	***t***	***p*-Value**
BPFS-AI	−0.005	−0.037	0.970	0.083	0.612	0.544	0.038	0.278	0.782
BPFS-IPs	0.005	0.036	0.971	−0.193	−1.491	0.142	−0.159	−1.200	0.236
BPFS-NRs	−0.061	−0.460	0.647	0.010	0.078	0.939	0.013	0.092	0.927
BPFS-SH	−0.181	−1.327	0.191	0.194	1.419	0.162	0.186	1.388	0.187
BPFS-TOT	−0.079	−0.592	0.557	0.033	0.242	0.810	0.029	0.210	0.835

Note: significant *p*-values (*p* < 0.05) are indicated in **bold**. AI—affective instability; BD—youth with pediatric bipolar disorder; BD-OFF—offspring of subjects with BD; BPFS—Borderline Personality Features Scale; IPs—identity problems; NRs—negative relationships; SH—self-harm; TOT—total.

**Table 5:** Linear regressions of BPFS subscales and total scores and Cambridge gambling task in 39 youth with BD and 18 BD-OFF.

	CGT-Delay Aversion			CGT-Deliberation Time		CGT-Overall Proportion Bet			
	β	*t*	*p*-Value	β	*t*	*p*-Value	β	*t*	*p*-Value
BPFS-AI	−0.014	−0.095	0.925	0.093	0.694	0.491	0.119	0.809	0.422
BPFS-IPs	−0.389	−2.920	**0.005**	0.186	1.476	0.147	0.367	2.766	**0.008**
BPFS-NRs	−0.205	−1.426	0.161	0.213	1.671	0.101	0.236	1.676	0.100
BPFS-SH	−0.189	−1.262	0.213	0.237	1.800	0.078	0.212	1.439	0.157
BPFS-TOT	−0.247	−1.723	0.092	0.228	1.776	0.082	0.289	2.058	**0.045**
	**CGT-Quality of Decision Making**			**CGT-Risk Adjustment**		**CGT-Risk Taking**			
	**β**	***t***	***p*-Value**	**β**	***t***	***p*-Value**	**β**	***t***	***p*-Value**
BPFS-AI	−0.242	−1.638	0.108	−0.155	−1.057	0.296	0.134	0.901	0.372
BPFS-IPs	−0.066	−0.451	0.654	−0.102	−0.719	0.476	0.366	2.732	**0.009**
BPFS-NRs	−0.235	−1.634	0.109	−0.218	−1.544	0.129	0.253	1.788	0.080
BPFS-SH	−0.335	−2.298	**0.026**	−0.226	−1.538	0.131	0.243	1.641	0.107
BPFS-TOT	−0.271	−1.878	0.067	−0.217	−1.519	0.135	0.308	2.188	**0.034**

Note: significant *p*-values (*p* < 0.05) are indicated in **bold**. AI—affective instability; BD—youth with pediatric bipolar disorder; BD-OFF—offspring of subjects with BD; BPFS—Borderline Personality Features Scale; CGT—Cambridge gambling task; IPs—identity problems; NRs—negative relationships; SH—self-harm; TOT—total.

**Table 6:** Linear regressions of BPFS subscales and total scores and RVP in 39 youth with BD and 18 BD-OFF.

	RVPA			RVP-Mean Latency		
	β	*t*	*p*-Value	β	*t*	*p*-Value
BPFS-AI	−0.052	−0.312	0.757	0.017	0.114	0.910
BPFS-IPs	−0.057	−0.361	0.720	0.150	1.075	0.290
BPFS-NRs	−0.079	−0.499	0.621	0.106	0.754	0.456
BPFS-SH	−0.037	−0.211	0.834	0.173	1.136	0.264
BPFS-TOT	−0.071	−0.435	0.667	0.141	0.973	0.337
	**RVP-Probability of False Alarm**			**RVP-Probability of Hit**		
	**β**	***t***	***p*-Value**	**β**	***t***	***p*-Value**
BPFS-AI	−0.014	−0.088	0.930	−0.071	−0.425	0.673
BPFS-IPs	0.075	0.498	0.622	−0.056	−0.357	0.724
BPFS-NRs	0.100	0.661	0.513	0.050	0.314	0.756
BPFS-SH	0.034	0.206	0.838	−0.040	−0.232	0.818
BPFS-TOT	0.062	0.395	0.695	−0.035	−0.211	0.834

Note: significant *p*-values (*p* < 0.05) are indicated in **bold**. AI—affective instability; BD—youth with pediatric bipolar disorder; BD-OFF—offspring of subjects with BD; BPFS—Borderline Personality Features Scale; IPs—identity problems; NRs—negative relationships; RVP—rapid visual processing; SH—self-harm; TOT—total.

**Table 7:** Linear regressions of BPFS subscales and total scores and SOC in 39 youth with BD, 18 BD-OFF, and 50 HCs.

SOC-Moves			
	β	*t*	*p*-Value
BPFS-AI	−0.190	−1.418	0.162
BPFS-IPs	0.006	0.050	0.960
BPFS-NRs	−0.084	−0.650	0.519
BPFS-SH	0.009	0.067	0.947
BPFS-total	−0.074	−0.559	0.578

Note: significant *p*-values (*p* < 0.05) are indicated in **bold**. AI—affective instability; BD—youth with pediatric bipolar disorder; BD-OFF—offspring of subjects with BD; BPFS—Borderline Personality Features Scale; HCs—healthy controls; IPs—identity problems; NRs—negative relationships; SH—self-harm; SOC—stockings of Cambridge.

## 4. Discussion

The results can be summarized as follows: youth with BD and BD-OFF showed greater levels of affective instability, negative relationships, and self-harm compared to HCs. These findings regarding borderline personality features, namely, greater self-harm, affective instability, an unstable perception of the self, and interpersonal problems, in youth with BD and BD-OFF are in line with studies showing a higher prevalence of borderline personality features in adults with BD [42] and BD-OFF [43].

Youth with BD showed increased latency for decision making and reduced problem-solving abilities in comparison to HCs. The results for CGT-deliberation time and the poorer number of problems solved in the minimum number of moves corroborate the evidence of executive dysfunction in BD, especially in planning, strategy formation, and decision making. Several studies report slower decision making in BD compared with HCs, in both manic [44,45] and euthymic states [46]. Also, significant deficits in strategy development and problem-solving [47] have been reported during acute episodes, which also persist during periods of remission [48].

However, youth with BD did not show deficits in affective regulation. Furthermore, no cognitive and affective differences were found between BD-OFF and HCs. These latter findings partially contradict the previous literature on this population [49,50,51]. The small sample size of BD-OFF (N = 18) may have limited the reliability of results found, and therefore, larger sample sizes are needed to investigate cognitive and affective alterations in BD-OFF.

Identity disturbance refers to a markedly and persistently unstable self-image or sense of self [52]. A strong sense of self helps an individual make good decisions and adapt to changes, whereas an unstable sense of self may result in the development of nonproductive coping strategies, including avoidance and self-blaming [53,54]. These strategies have been related to high levels of impulsivity, risk taking, and a preference for an immediate reward [55,56]. In this study, linear regressions demonstrated that having greater identity problems, a feature of BPD, was associated with difficulty postponing immediate rewards, delaying gratification, and increased risk taking in youth with BD and BD-OFF. These findings are in line with those from the meta-analysis of Paret and colleagues [57], in which individuals with BPD discounted delayed rewards more strongly and achieved lower net gains in the Iowa gambling task (IGT) when compared to HCs. Additionally, Bazanis et al. [58] reported impulsive responsiveness in individuals with BPD who completed a computerized decision-making task.

In youth with BD and BD-OFF, an increased severity of self-harm correlated with poorer decision making. This correlation is in line with the available evidence in individuals with or without BPD [59,60,61,62,63]. In a study by Dimick et al. [60], youth with BD and no self-harm differed in the CGT task from youth with BD and self-harm behaviors, and healthy controls. Notably, youth with BD and no self-harm behaviors had greater self-control and better decision making than the other two groups. As Dimick et al. [60] suggest, this finding may be a learned adaptive response to adverse experiences. Importantly, the finding that BD youth with no self-harm behaviors made less risky decisions needs to be further explored by investigating the neurobiology of BD in youth, in order to clearly identify those at a high risk for self-harm.

In the present study, the negative relationship and self-harm subscales were linked to reduced response times for positive words (i.e., a positive bias) in both youth with BD and BD-OFF. Prior studies indicate that individuals with BPD tend to show heightened sensitivity to negative cues [64,65,66] and struggle to interpret positive cues accurately [56,67]. However, these earlier studies did not assess the potential influence of co-occurring BD. Unlike individuals with BPD, those with BD demonstrate a mood-congruent attentional bias, marked by increased focus on positive information, while individuals experiencing depression show quicker processing of negative content [68,69,70]. This mood-aligned attentional pattern seems to extend to BD-OFF as well. Kærsgaard et al. [71] reported elevated responsiveness to positive social information in twins with a family history of BD and proposed that this positive bias might be an endophenotypic marker of BD. Thus, multiple interacting features may emerge when BD and BPD are comorbid. Although past research supports the idea that individuals with BPD tend to react impulsively to negative emotion [61], the current findings suggest a potentially more nuanced link between self-harm and positive bias. Additionally, self-harm behaviors have been tied to the pursuit of intense emotional experiences to counter distress and heightened reward sensitivity, which could explain the quicker responses to positive stimuli [72,73].

All the aforementioned correlations were observed in both BD and BD-OFF, with no emerging group interaction effect. These findings reinforce the concept of a possible shared alteration in cognitive and behavioral substrates in BD and BD-OFF [74,75,76]. Moreover, they corroborate the available evidence of common pathophysiological mechanisms in these disorders [77,78,79,80]. Such mechanisms appear to involve deficits in decision making, risk taking, emotional processing, identity problems, and self-harm. Further studies are needed to corroborate the relationship between BD and BPD in youth with BD and BD-OFF.

### Limitations

This study has several limitations. First, the small sample size reduces the statistical power and generalizability of the results, which need to be confirmed in larger samples. Second, the limited sample size restricted our ability to subdivide the youth into groups based on BD and BD-OFF diagnosis with or without BPD comorbidity. Comparisons among the resulting four groups (i.e., youth with BD and comorbid BPD, youth with BD without BPD, BD-OFF with BPD, and BD-OFF without BPD) would have provided additional insights beyond the dimensional analyses conducted in the present study. Therefore, future studies with larger sample sizes are warranted. Third, we investigated only a limited range of cognitive aspects. A more comprehensive cognitive assessment is needed to clarify our findings. Importantly, impaired memory and learning processes are thought to play a central role in altered attention and affective responsiveness in individuals with BPD [81,82,83,84]. An examination of memory and learning might offer insight into certain aspects of our results, such as the relationship among self-harm, negative relationships, and a shorter response latency for positive words. Fourth, the cross-sectional design does not allow us to assess changes over time or to clearly establish causal relationships amongst our findings. For instance, factors such as previous positive or negative experiences and the course of BD may have influenced our results. Notably, a longitudinal study design might clarify the significance of our findings in BD-OFF. Additionally, the cross-sectional nature of this study and the age of the participants prevent the analysis of possible age-related changes in borderline personality disorder features. Fifth, although the results were corrected for the effect of medication, the small sample size limited this investigation to classes only. Since medications can influence behavior and mood [85,86], a deeper understanding of the assumed effect of medications would clarify the impact of borderline features and BD on cognition.

## 5. Conclusions

Borderline personality features are associated with cognitive and affective alterations in youth with BD and BD-OFF. These findings warrant attention on the onset and progression of BPD features in youth with BD. The significance of this lies in early treatment strategies to target BPD features not only in youth with BD but also in those at risk of developing BD. Such targeted interventions could help reduce symptom severity and improve functional outcomes, potentially reducing the clinical burden of BD. Further studies are required to understand the relationship between BD, BD-OFF, and BPD and their shared cognitive and affective alterations.

## Figures and Tables

**Table 1:** Demographic and clinical characteristics of 39 youth with BD, 18 BD-OFF, and 50 HCs.

		BD (N = 39)	BD-OFF (N = 18)	HCs (N = 50)	F or χ^2^	*p*-Value
Demographics						
	Age (y), mean ± SD	12.83 ± 3.22	12.37 ± 2.79	12.08 ± 3.06	0.65	0.524
	Sex, n (%)				0.35	0.841
	Female	22 (56.4)	9 (50.0)	29 (58.0)		
	Male	17 (43.6)	9 (50.0)	21 (42.0)		
	Years of education, mean ± SD	7.08 ± 2.96	4.64 ± 3.26	6.33 ± 2.90	11.38	**<0.001**
	Race, n (%)				9.79	0.134
	Asian	0 (0)	0 (0)	3 (6)		
	African American	7 (17.9)	2 (11.1)	14 (28.0)		
	Caucasian	29 (74.4)	15 (83.3)	33 (66.0)		
	Other	3 (7.7)	1 (5.6)	0 (0)		
	Ethnicity, n (%)				0.37	0.830
	Hispanic	8 (20.5)	5 (27.8)	11 (22.4)		
	Non-Hispanic	31 (79.5)	13 (72.2)	38 (77.6)		
	I.Q., mean ± SD	105.17 ± 13.46	91.27 ± 10.23	103.19 ± 14.01	6.09	**0.003**
Clinical						
	CDRS, mean ± SD	54.97 ± 11.59	44.81 ± 12.69	32.59 ± 5.66	59.82	**<0.001**
	YMRS, mean ± SD	15.10 ± 10.53	4.69 ± 3.81	3.08 ± 3.07	34.39	**<0.001**
	BD type, n (%)					
	Type 1	29 (74.4)	0 (0)	0 (0)	-	-
	Type II	2 (5.1)	0 (0)	0 (0)	-	-
	Not otherwise specified	8 (20.5)	0 (0)	0 (0)	-	-
	Comorbidity, n (%)					
	None	10 (26.3)	12 (66.7)	50 (100)	53.82	**<0.001**
	MDD	0 (0)	2 (11.1)	0 (0)	10.08	**0.006**
	ADHD	24 (61.5)	4 (22.2)	0 (0)	43.12	**<0.001**
	OCD	5 (13.2)	0 (0)	0 (0)	9.39	**0.009**
	Panic Disorder	2 (5.1)	0 (0)	0 (0)	3.55	0.169
	GAD	10 (25.6)	2 (11.1)	0 (0)	14.47	**<0.001**
	Current pharmacotherapy, n (%)					
	ADs	16 (41.0)	4 (22.2)	0 (0)	24.44	**<0.001**
	APs	27 (69.2)	4 (22.2)	0 (0)	51.51	**<0.001**
	MSs	12 (30.8)	1 (5.6)	0 (0)	20.32	**<0.001**
	Stimulants	19 (48.7)	20 (18.7)	0 (0)	36.67	**<0.001**
	BDZs	0 (0)	0 (0)	0 (0)	-	-

Note: significant *p*-values (*p* < 0.05) are indicated in **bold**. ADs—antidepressants; ADHD—attention-deficit hyperactivity disorder; APs—antipsychotics; BD—youth with bipolar disorder; BD-OFF—offspring of subject with BD; BDZs—benzodiazepines; CDRS—Children’s Depression Rating Scale; GAD—generalized anxiety disorder; HC(s)—healthy control(s); I.Q.—intelligence quotient; MDD—major depressive disorder; MSs—mood stabilizers; n—number; OCD—obsessive–compulsive disorder; SD—standard deviation; y—year(s); YMRS—Young Mania Rating Scale.

## Data Availability

The raw data supporting the conclusions of this article will be made available by the authors on request. The data are not publicly available due to privacy restrictions.

## Abbreviations

The following abbreviations are used in this manuscript:

BD	Bipolar Disorder
BD-OFF	BD-Offspring
BDP	Borderline Disorder Personality
HC(s)	Healthy Control(s)
BPFS-C	Borderline Personality Features Scale for Children
CANTAB	Cambridge Neuropsychological Test Automated Battery
CGT	Cambridge Gambling Task
SOC	Stockings of Cambridge
AGN	Affective Go/No-Go
RVP	Rapid Visual Processing
ANOVAs	Analysis of Variance
DSM	Diagnostic and Statistical Manual of Mental Disorders
ADHD	Attention-Deficit Hyperactivity Disorder
BD-NOS	BD-Not Otherwise Specified
COBY	Course and Outcome of Bipolar Youth
MINI-KID	Mini International Neuropsychiatric Interview for Children and Adolescents
WASI-II	Wechsler Abbreviated Scale of Intelligence–Second Edition
CDRS-R	Children’s Depression Rating Scale–Revised
YMRS	Young Mania Rating Scale
IQ	Intelligence Quotient
